# Effect of genetic background and delivery route on the preclinical properties of a live attenuated RSV vaccine

**DOI:** 10.1371/journal.pone.0199452

**Published:** 2018-06-19

**Authors:** Rachel Groppo, Joshua DiNapoli, Kwang Il Jeong, Michael Kishko, Nicholas Jackson, Harold Kleanthous, Simon Delagrave, Linong Zhang, Mark Parrington

**Affiliations:** 1 Research and non-clinical safety, Sanofi Pasteur, Cambridge, Massachusetts, United States of America; 2 FluNeXt, Sanofi Pasteur, Cambridge, Massachusetts, United States of America; 3 External R&D, Sanofi Pasteur, Cambridge, Massachusetts, United States of America; University of Iowa, UNITED STATES

## Abstract

A safe and effective vaccine against RSV remains an important unmet public health need. Intranasally (IN) delivered live-attenuated vaccines represent the most extensively studied approach for immunization of RSV-naïve infants and children, however, achieving an effective balance of attenuation and immunogenicity has proven challenging. Here we report pre-clinical immunogenicity and efficacy data utilizing a live-attenuated vaccine candidate, RGΔM2-2, which was obtained by deleting the M2-2 open reading frame from the genome of the MSA1 clinical isolate. Intramuscular (IM) administration of RGΔM2-2 in cotton rats induced immunity and protective efficacy that was comparable to that induced by intranasal (IN) immunization. In contrast, the protective efficacy of RGΔM2-2 delivered by the IM route to African green monkeys was substantially reduced as compared to the efficacy following IN administration, despite comparable levels of serum neutralizing antibodies. This result suggests that mucosal immunity may play an important role in RSV protection. The RGΔM2-2 vaccine also demonstrated different attenuation profiles when tested in cotton rats, non-human primates, and a human airway epithelial (HAE) cell model. The data suggest RGΔM2-2 is less attenuated than a similarly designed vaccine candidate constructed on the A2 genetic background. These findings have important implications with regard to both the design and the preclinical safety testing of live-attenuated vaccines.

## Introduction

Respiratory syncytial virus (RSV) is a common and very contagious virus that infects the respiratory tract of infants and young children. In the United States, RSV is the leading cause of hospitalization in children less than 1 year of age [1] and is associated with a considerable burden of emergency room and outpatient care, with 10% of children less than 5 years of age receiving medical attention for RSV-associated illness each year [2]. Worldwide, RSV causes 3 million hospitalizations and 265,000 deaths yearly in children less than 5 years of age [3]. While the prophylactic antibody Synagis is administered to at risk infants, there is currently no licensed vaccine [4]. Given the worldwide prevalence of RSV and high disease burden, an effective vaccine for this virus is considered a priority.

A RSV live-attenuated vaccine approach is generally favored for infants to avoid vaccine enhanced disease as was seen in clinical trials involving the use of formalin-inactivated RSV [5]. In addition, live-attenuated RSV vaccines would be administered intranasally (IN), offering the greatest promise of inducing both mucosal and systemic immunity for the protection of young infants [6]. The most clinically advanced infant RSV vaccine candidates consist of live-attenuated RSV viruses administered IN [7]. As these vaccines are administered via the natural route of infection, they must achieve the correct balance of attenuation and immunogenicity. Different iterations of these vaccines have been tested for the past 40 years [8–10]. One of the most advanced vaccine candidates is RSV MEDI ΔM22, where the M2-2 gene of the RSV A2 strain has been deleted leading to attenuation. This vaccine was able to induce serum neutralizing titers of 1:97 and had a favorable attenuation profile in naïve infants [11]. A key advantage of this design is the increase in antigen expression due to an up-regulation of gene transcription caused by the deletion of M2-2 [11,12]. Since a large deletion is responsible for this vaccine’s attenuation profile, reversion to wildtype is unlikely [11].

Though live-attenuated RSV vaccine by IN immunization is the main vaccination strategy for young infants, achievement of the balance between attenuation and immunogenicity has been challenging. Attenuation generally reduces immunogenicity, due in part to lower antigen expression associated with reduced viral replication. Candidate vaccines evaluated during the 1960s-1990s were either insufficiently attenuated [9,13] or over attenuated [14]. As an alternative vaccination strategy to mitigate the risk of insufficient attenuation, we investigated intramuscular (IM) immunization of live-attenuated RSV vaccines. Six clinical trials with live RSV, parenteral, single injection of 10^3.2^–10^3.9^ TCID_50_ have been reported [15–17]. Most children developed antibody following vaccination with no evidence of disease enhancement after exposure to natural RSV infection. However, clinical efficacy was not demonstrated. The lack of efficacy could be due to the low dose and single administration, therefore higher and/or additional doses may resolve this efficacy issue. One key advantage of RSV IM immunization is the decoupling of the attenuation/immunogenicity balance necessitated by IN immunization. Thus high doses of RSV can be given IM, boosting serum antibody responses in infants, a population in which a vaccine typically does not induce high antibody responses particularly after a single immunization [5]. To investigate IM immunization, we chose to take advantage of the well documented ΔM2-2 attenuation profile and create a vaccine strain RGΔM2-2 with a deletion of the M2-2 gene based on our proprietary clinical isolate MSA1. Subsequently, studies of IM and IN immunizations of RGΔM2-2 in cotton rats and non-human primates (NHP) were carried out. Although IM immunization of RGΔM2-2 induced comparable immunity as IN immunization, it only conferred partial protection while the IN route showed full protective efficacy, highlighting the importance of mucosal immunity in protection.

## Materials and methods

### Cells and virus

Vero CCL 81.2 cells were obtained from ATCC. RSV strains A2 and Long were purchased from ATCC. RSV strain MSA1 was derived by rescue of infectious virus from a cDNA clone of a proprietary clinical isolate as described [18]. RGΔM2-2 was created by maintaining the entire M2-1 gene and deleting the M2-2 open reading frame following M2-1 in a MSA1 background. Infectious RGΔM2-2 was rescued similarly as MSA1. Infectious virus was amplified in Vero cells. Reporter virus strains expressing luciferase were constructed and recovered similarly. Growth comparison of MSA1 and RGΔM2-2 was performed in Vero cells, infected at an MOI = 0.001 and incubated at 37°C. At the day of harvest, cells were scraped into the media and frozen. Titration was essentially identical to brochoalveolar lavage titration as described below. Human airway epithelial cells were obtained from Lonza Walkersville, Inc. Culture and infection of these cells was performed as described [18].

### Infection of cotton rats with RSV expressing luciferase

**Fig 1:**
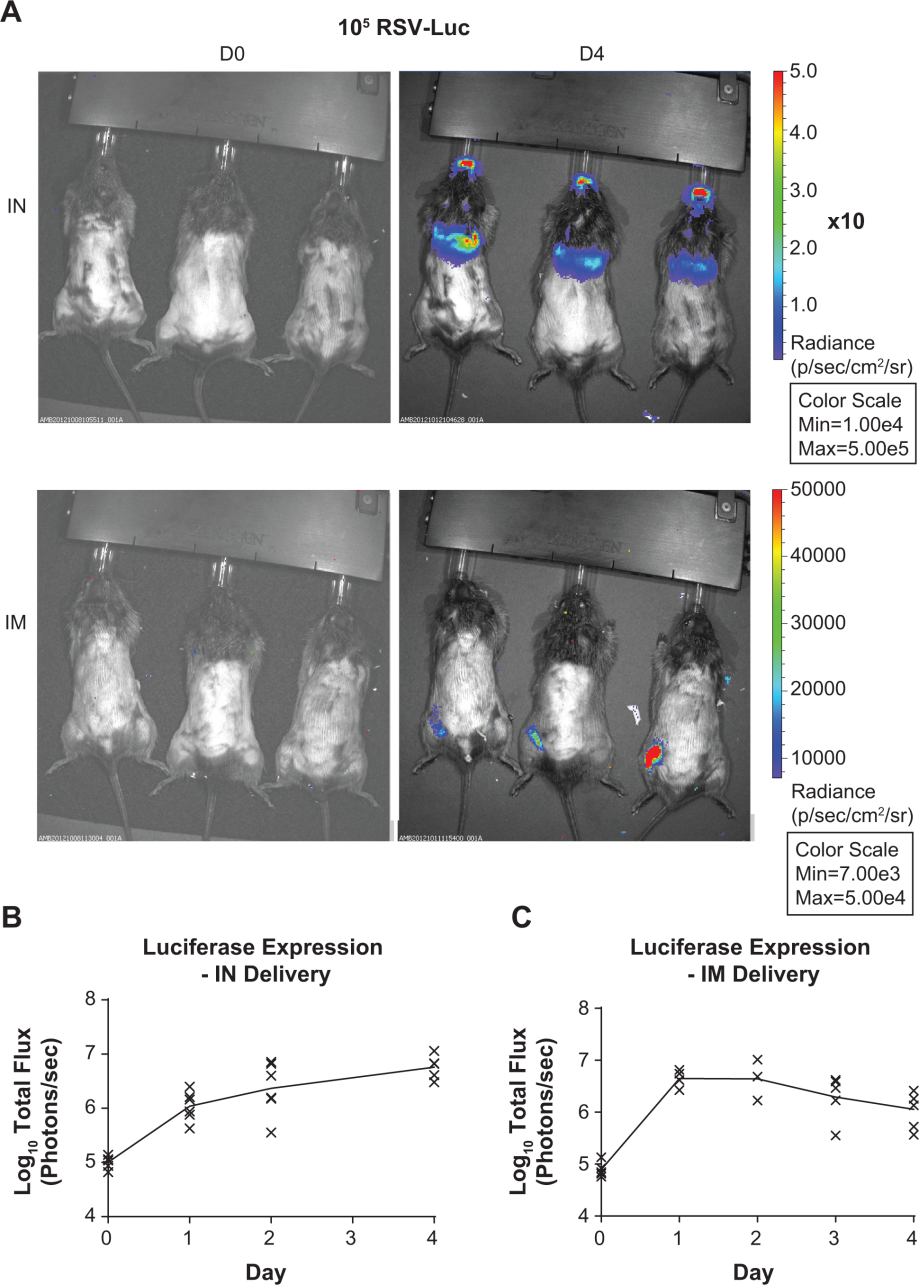
Infection of cotton rats with a recombinant RSV expressing luciferase. Cotton rats were infected with a recombinant RSV expressing luciferase either IN or IM. Animals were imaged daily for luciferase expression. (A) Representative image of luciferase expression on D4 post inoculation. (B) Quantitation of luciferase expression in animals immunized IN. (C) Quantitation of luciferase expression in IM immunized animals.

7–9 week old inbred female cotton rats (*Sigmodon hispidus*) were obtained from Harlan (now EnVigo). This study was carried out in accordance with the recommendations in the Guide for the Care and Use of Laboratory Animals of the National Institutes of Health. The protocol was approved by the Committee on the Ethics of Animal Experiments of Sanofi Pasteur (protocol 2012-05-01). All efforts were made to minimize suffering including anesthesia via isoflurane. Animals were housed in standard rat cages and were fed a standard diet of rodent chow and water. Cotton rats were divided into 4 groups. Animals were briefly anesthetized with isoflurane and then were administered 100 μl of the indicated inoculum by the IM or IN route. For IM inoculation, the entire volume was administered to the left quadriceps muscle using a 5/8” 27 gauge needle. For IN inoculation, 50 μl was administered to each nostril using a pipette. The RSV-luc preparation used for infection in this study had been purified over a sucrose gradient to remove exogenous luciferase present in the tissue culture supernatant and was diluted to a concentration of 5.4 x 10^6^ PFU/ml in SFM4 MegaVir medium (HyClone) containing 2mM L-glutamine and 1% antibiotic/antimycotic. Heat inactivation was accomplished by heating virus samples to 56°C for 40 min prior to administration. At imaging time points (0 hours post-infection (HPI), 24 HPI, 48 HPI, 72 HPI, 96 HPI), animals received 500 μl of 150 mg/ml Viviglo Luciferin (Promega) by the intraperitoneal route while under isoflurane anesthesia. They were then imaged using an IVIS Spectrum imager (Caliper LifeSciences) on the “small binning” setting. Images were analyzed using the Perkin Elmer Living Image software. We found that several animals had intermittent absence of luciferase signal at time points over the course of the experiment owing to technical difficulties during luciferin delivery. These data points were excluded from the analysis in Fig 1 (9 of 60 data points excluded total).

### Immunization and infection of cotton rats

Cotton rat studies were conducted at Sigmovir Biosystems, Inc., Rockville, MD, USA, in accordance with the National Institutes of Health guidelines and the facility’s Institutional Animal Care and Use Committee’s approved study protocol #15. Briefly, 6–8 week old inbred female cotton rats (*Sigmodon hispidus*) were obtained from a colony maintained by Sigmovir. Animals were housed in standard rat cages and were fed a standard diet of rodent chow and water. The colony was demonstrated to be free of antibodies against paramyxoviruses prior to their inclusion in this study. For infection or immunization, the viruses used were first diluted to an appropriate working concentration in cold PBS. Animals were lightly anesthetized with isoflurane prior to intranasal or intramuscular immunization. Intranasal infection was performed using a total volume of 100 μl (~50 μl per nostril). Intramuscular immunizations were also done in a final volume of 100 μl delivered to the right thigh muscle. To evaluate pulmonary virus replication in challenged animals, animals were sacrificed by carbon dioxide asphyxiation on day 4 post-infection and the lungs were harvested. Homogenization medium was prepared by supplementing Hank’s Balanced Salt Solution (HBSS) with 0.218 M sucrose, 4.4 mM glutamate, 3.8 mM KH_2_PO_4_, and 7.2 mM K_2_HPO_4_. Lungs were placed in an appropriate volume of homogenization medium to yield a 10% w/v solution and homogenization was done using a Tenbroeck tissue homogenizer. Lung homogenates were cleared by centrifugation and stored at -80° C prior to titration. To determine virus content in lung samples, serial dilutions of each homogenate were prepared in Eagle’s minimal essential medium (EMEM) supplemented with Earle’s balanced salt solution. Confluent monolayers of HEp-2 cells were infected in triplicate with each dilution for 1 hour, after which the inoculum was discarded and overlay consisting of EMEM supplemented with 0.75% methyl cellulose, 1% fetal bovine serum, 2 mM L-glutamine, 0.05 mg/L gentamicin, and 0.25 μg/L Fungizone was added. On day 4 post-infection, the overlay medium was discarded and plaques were stained using 0.07% crystal violet. The titer for a given sample was based on the dilution resulting in 10–100 plaques when possible, and the reported value represents the mean plaque count of all three replicate wells from that dilution. Samples with undetectable plaques were assigned a value at the limit of detection.

### Immunization and infection of primates

The African Green Monkeys (AGM) (*Chlorocebus aethiops*) used in this study were obtained from St. Kitts Island via suppliers with free range breeding groups. Permits obtained for these animals included: US Fish and Wildlife Service Declaration for Importation (LE062441-1) and CITES (646/2012). AGM were prescreened by ELISA to RSV F protein. All AGM procedures were carried out at Bioqual (Rockville, MD) in accordance with the recommendations in the Guide for the Care and Use of Laboratory Animals of the National Institutes of Health. Protocols 13-3551-07 and 13-3551-58 were approved by the Bioqual Institutional Animal Care and Use Committee. When needed, sedation was induced by intramuscular injection of 10–15 mg/kg of ketamine. Animals were housed in 6.0 bio containment caging with 10–15 air changes. The staff changed personal protective equipment between each group to prevent cross contamination. Animals were on a 12 hour light cycle with feeding once a day also with daily fruit. Animals were provided with environmental enrichment. Cages were cleaned and checked daily with two health observations completed daily by Bioqual technicians. After the conclusion of the study, animals were returned to a recycle pool.

For attenuation studies, AGMs were inoculated with 2x10^5.5^ PFU RSV via an intranasal and intratracheal (IN/IT) route, 1ml per site. For immunogenicity and challenge studies, AGMs were either immunized similarly IN/IT with 1x10^5.5^ PFU of the indicated RSV strain or intramuscularly into the right thigh, 1ml total volume. When challenged, RSV strain A2 was administered IN/IT, 2x10^6^ PFU total, 1ml per site. Brochoalveolar lavage samples (BAL) were taken days 3, 5, 7, and 10 days post challenge. For BAL sampling, 10ml Hank’s balanced salt solution (HBSS) was administered to the lung area via sterile catheter and syringe. 1/10^th^ volume 10x SPG (2.18 M sucrose, 0.038 M KH_2_PO_4_, 0.072 M K_2_HPO_4_, 0.06 M L-glutamine at pH 7.1) was added to recovered samples, mixed, aliquoted and snap frozen on dry ice.

For quantitation of RSV, BAL samples were serially diluted in DMEM/1x L-glutamine and added to 24-well plates of Vero CCL 81.2 cells. Binding was carried out at 37°C/5% CO_2_ with occasional rocking. After one hour, 0.75% methyl cellulose/DMEM/2% FBS/2% L-glutamine/2% antibiotic-antimycotic was added and plates were incubated for 5 days. Subsequently, methyl cellulose was removed, cells fixed with methanol and anti-RSV antibody conjugated to horse radish peroxidase (Abcam AB20686) was used to visualize plaques. Titers are expressed as plaque forming units per ml (PFU/ml).

### Measurement of immune responses

RSV ELISA assays were performed by coating 96 well ELISA plates with a proprietary formulation of RSV F protein, 50ng/well. After overnight incubation at 4°C, plates were washed and blocked with blocking solution (KPL 50-61-00). Test serum was serially diluted in blocking solution and added to ELISA plates for 1hr at 37°C. Plates were washed and appropriate horse radish peroxidase conjugated secondary antibody was added. Following extensive washing, TMB peroxidase substrate (KPL) was added for 20 minutes and the reaction stopped. The absorbance at 450nm was measured.

RSV neutralization titers were determined on Vero CCL 81.2 cells against RSV Long strain. Heat inactivated serum was serially diluted with DMEM/1x glutamine/2% FBS. Hyper-immune mouse serum was used as a positive control. Guinea pig complement (Cedar lane labs) was used at 5% final concentration. Plaque formation was quantitated as described above. The 60% plaque reduction neutralization titer (PRNT) was determined.

## Results

### RSV mediates robust de novo protein synthesis without productive replication following IM delivery in cotton rats

We used a recombinant RSV expressing the luciferase gene as described in Methods, which allowed us to track luciferase expression over time *in vivo* using the cotton rat model. Robust luciferase expression was observed in all cotton rats that received the RSV-luc virus by the IM or IN route for at least 4 days post-immunization (Fig 1A). Luciferase expression was still climbing by day 4 following IN delivery (Fig 1B), which is consistent with past data showing RSV replication peaks near day 4 in this model [19]. In contrast, IM delivery in cotton rats resulted in peak luciferase expression days 1–2, though signal remained strongly detectable until 4 days post-immunization (Fig 1C). In addition, we did not see evidence of virus replication at any sites beyond the site of administration (as indicated by luciferase protein expression), consistent with a previous report [20]. This result suggested IM immunization did not result in productive replication, which is an important safety consideration for systemic delivery of a replication competent virus. Taken together, the cotton rat model suggests that IM delivery of RSV is a safe and effective route of immunization owing to robust expression of viral antigens at the site of immunization while showing no evidence of systemic spread.

### The RGΔM2-2 virus is attenuated in cotton rats

**Fig 2:**
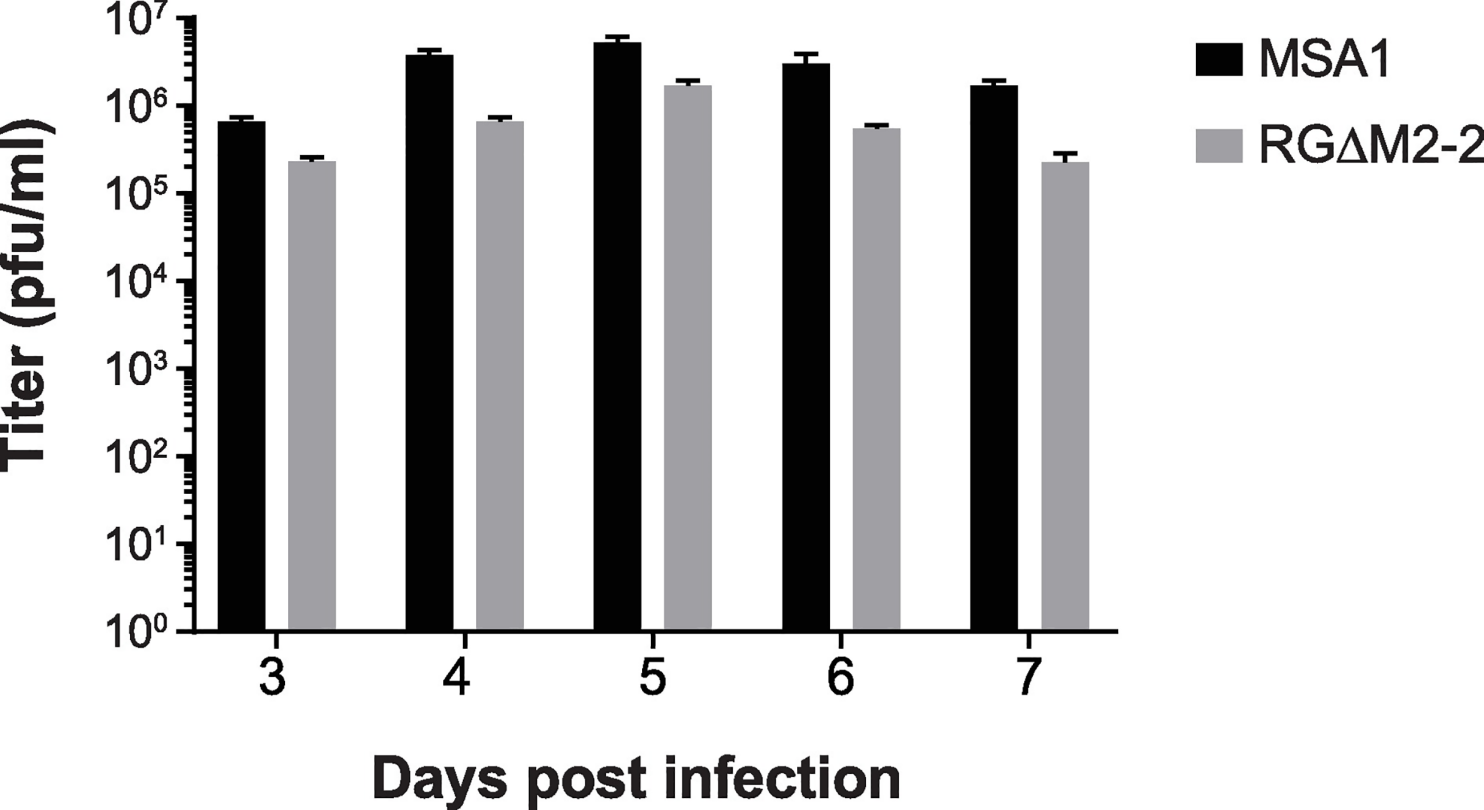
Growth of RGΔM2-2 versus parental virus in Vero cells. (A) Vero cells were infected at an MOI of 0.001 and incubated at 37°C. Samples harvested at the times indicated were titrated on Vero cells.

Though wildtype RSV could be used for IM vaccination, we felt it was important to generate a vaccine candidate that is attenuated in the lower respiratory tract (LRT) to address the hypothetical risk of respiratory tract exposure in the event of mishandling during a mass vaccination campaign. Previous studies showed deletion of the M2-2 gene in an A2-strain background caused an increase in protein expression and LRT attenuation, both desired phenotypes [11,12]. We therefore created a deletion of M2-2 within the proprietary RSV strain MSA1. A plasmid was synthesized containing the full cDNA of the MSA1 genome with the portion of the M2-2 gene that follows the stop codon of M2-1 deleted. After rescuing infectious virus, the *in vitro* growth of this RGΔM2-2 was compared to the parental MSA1 that was similarly rescued using reverse genetics. Yield of virus from Vero cells infected at an MOI of 0.001 showed RGΔM2-2 grew to slightly lower titers at all time points versus the parental MSA1 (Fig 2). Overall, the MSA1 virus achieved a peak titer of 5.3x10^6^ PFU/mL while that of RGΔM2-2 was 3x lower.

**Fig 3:**
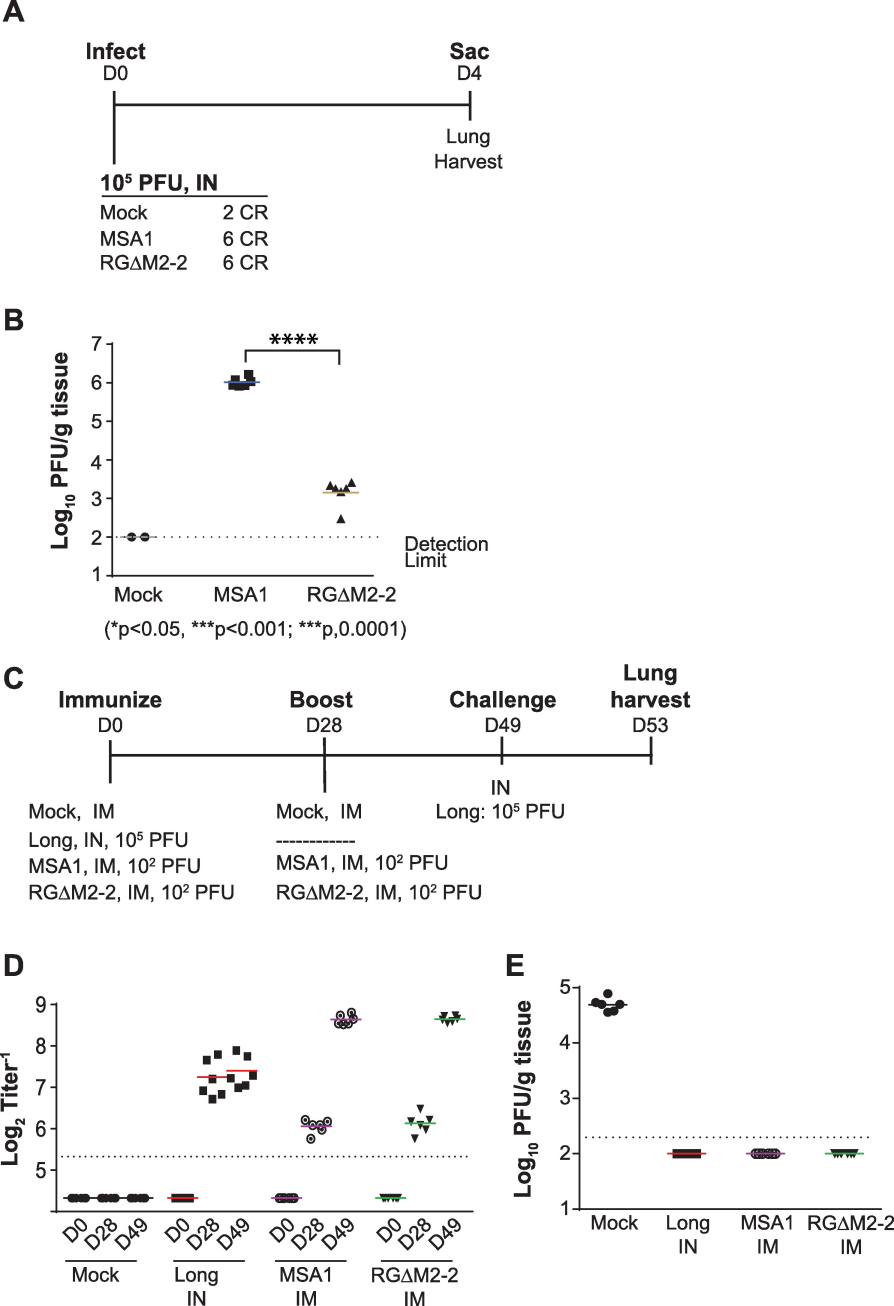
Attenuation and immunogenicity in cotton rats. (A) Groups of cotton rats were infected as indicated. (B) The degree of attenuation as amount of virus present in the lungs of animals in (A) was determined. ****p < 0.0001 by unpaired t test. (C) Immunogenicity study in cotton rats. Animals were immunized as indicated. (D) Serum neutralization titers were determined. (E) Protective efficacy as amount of challenge virus present in the lungs of immunized animals in (C).

The RGΔM2-2 vaccine candidate was then evaluated for LRT attenuation in the cotton rat model (Fig 3A). The cotton rat is highly permissive for RSV infection, and is commonly used for evaluation of RSV attenuation [21–24]. Indeed, we found that the deletion of the M2-2 open reading frame from the MSA1 RSV strain caused substantial attenuation of the virus (Fig 3B), consistent with past findings for an M2-2 deletion on an RSV A2 background [25]. Specifically, the M2-2 deletion resulted in a >2.5 log reduction in pulmonary viral load as compared to the parental strain. This might mitigate the hypothetical risk of IN exposure during a mass vaccination campaign with an IM vaccine candidate.

### The RGΔM2-2 virus is as immunogenic and efficacious as the parental virus following IM delivery in cotton rats

Given the substantial lower respiratory tract attenuation of the RGΔM2-2 virus in the cotton rat model, we sought to determine whether it would maintain its immunogenicity and efficacy following IM delivery. Animals were immunized and challenged as described in Fig 3C. RSV IN immunization using Long strain at a 10^5^ PFU dose was included as a positive control to represent a regimen that induces robust immunity and sterilizing protection against subsequent challenge based on past experience by our group and others [21,23]. Day 0, 28, and 49 serum samples were all evaluated for their ability to neutralize RSV Long strain in a complement-independent neutralization assay. All groups had a detectable neutralizing antibody response after the first immunization (Fig 3D). The RSV IN group had the highest titer of neutralizing antibodies after one immunization, achieving a titer of 7.2log_2_; however, it is notable that 1000-fold lower dose of either wild type virus or RGΔM2-2 by IM induced a strong neutralizing antibody response of 6.1log_2_ for both immunogens. This titer was previously associated with protection against RSV A infections in clinical trials [26]. Importantly, a second IM immunization with either the wild type or the RGΔM2-2 resulted in a ~6-fold increase, to a titer of 8.6log_2_ for both viruses. The potency of the response in immunized animals was confirmed by challenge with RSV Long strain (Fig 3E), which resulted in substantial pulmonary replication to a titer of 4.7log_10_ PFU/g in control animals, whereas all immunized groups were completely protected from virus replication in the lung. These findings suggest that RSV can be delivered intramuscularly to induce potent immunity comparable to that induced by IN immunization. Furthermore, deletion of the M2-2 gene, though highly attenuating, did not impact the immunogenicity or efficacy of parenterally delivered RSV.

### The RGΔM2-2 virus is not attenuated in primates or human airway epithelial cells

**Fig 4:**
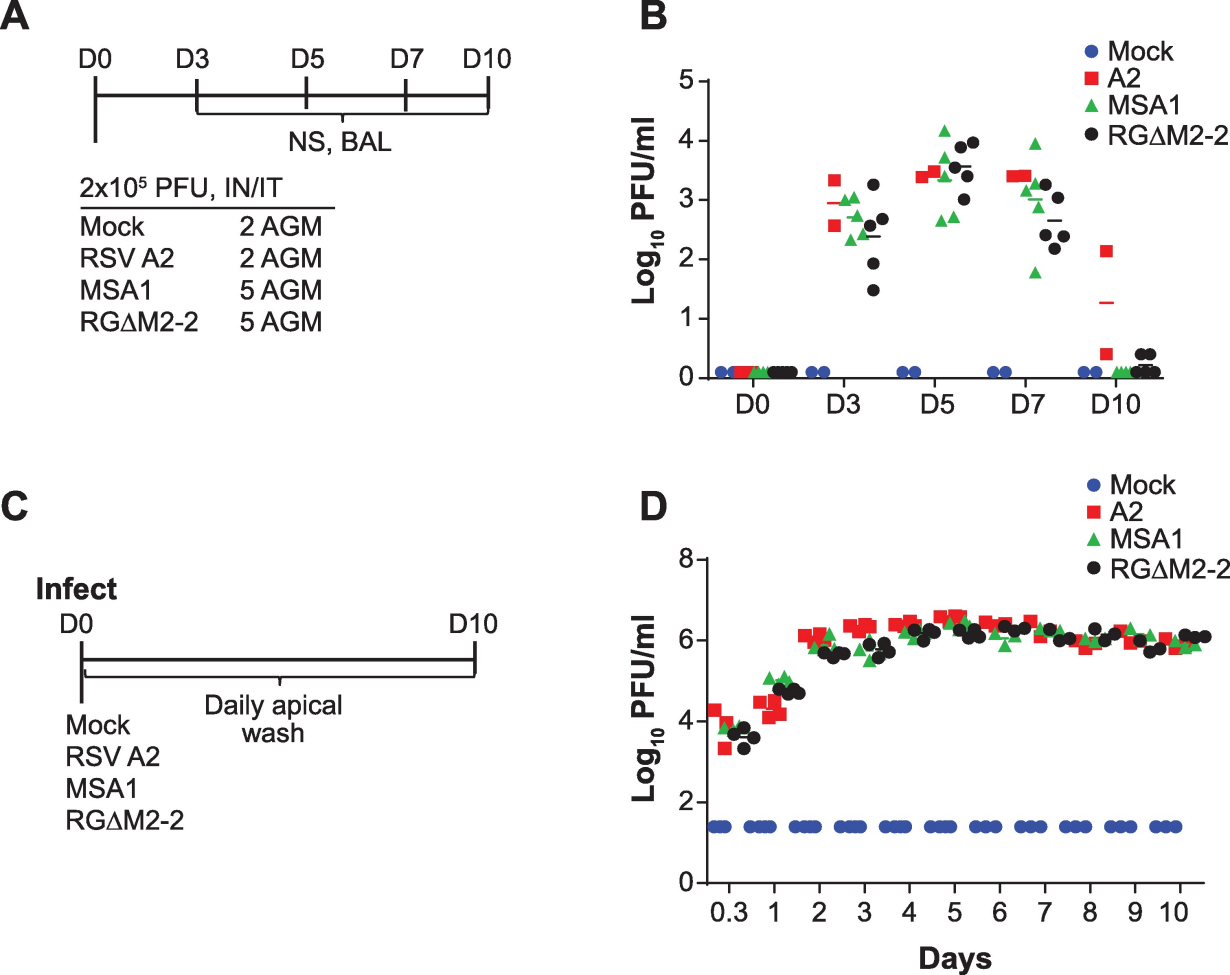
Attenuation profile in primates and HAE cells. (A) Primates were infected with viruses indicated. (B) BAL samples were harvested on days 3, 5, 7, and 10 post infection. The amount of virus present in these samples was determined. (C) HAE cells were infected as indicated and virus titers determined from apical wash samples taken daily (D). Statistical significance by unpaired t test was not observed for data in B and D.

To further assess the attenuation profile of the RGΔM2-2 virus, we inoculated groups of 5 African green monkeys (AGM) with either RGΔM2-2, mock or MSA1 virus (Fig 4A). Broncoalveolar lavage samples were taken at days 3, 5, 7, and 10 post infection and titrated. The lab RSV strain A2 was included for comparison purposes. Both wildtype viruses (A2 and MSA1) achieved peak titers of ~4log_10_ PFU/ml on days 5–7 post infection (Fig 4B), in agreement with previous studies [27]. In contrast to the pattern observed in cotton rats, BALs from primates infected with the vaccine strain RGΔM2-2 exhibited titers of virus similar to wildtype controls, peaking at D5 post infection with a titer of ~4log_10_ PFU/ml. Concurrently mock infected animals showed no presence of virus in the lung. Thus, RGΔM2-2 was not attenuated in primates.

To further characterize the attenuation profile of the RGΔM2-2 vaccine candidate, we utilized an *ex vitro* model of attenuation composed of differentiated, primary human airway epithelial (HAE) cells. This cell system has been shown to recapitulate many *in vivo* features of the human airway including a stratified cell layer, actively beating ciliated cells and mucous production [18,28,29]. Using this system, it has been demonstrated RSV binds to the CX3CR1 receptor on the surface of ciliated cells [29]. The HAE model has been used to characterize vaccine candidates [30,31].

Primary HAE cells from a 5 year old donor were expanded and differentiated. The HAE apical surface was infected with RSV strain A2, MSA1 or the vaccine candidate RGΔM2-2 (Fig 4C). Every day following infection, the apical surface of the cells was washed with PBS and the titer of virus in the wash determined on Vero cells. The overall attenuation profiles of each virus were similar to those observed in AGMs. Both of the wildtype viruses exhibited high levels of virus replication throughout the 10 day sampling period (Fig 4D). The RGΔM2-2 virus also showed high levels of virus replication with no apparent attenuation. Therefore, the RGΔM2-2 vaccine candidate exhibited different attenuation profiles in the separate attenuation models used; attenuated in cotton rats but not attenuated in primates or *in vitro* HAE cells.

### RGΔM2-2 immunogenicity and protective efficacy in African green monkeys

**Fig 5:**
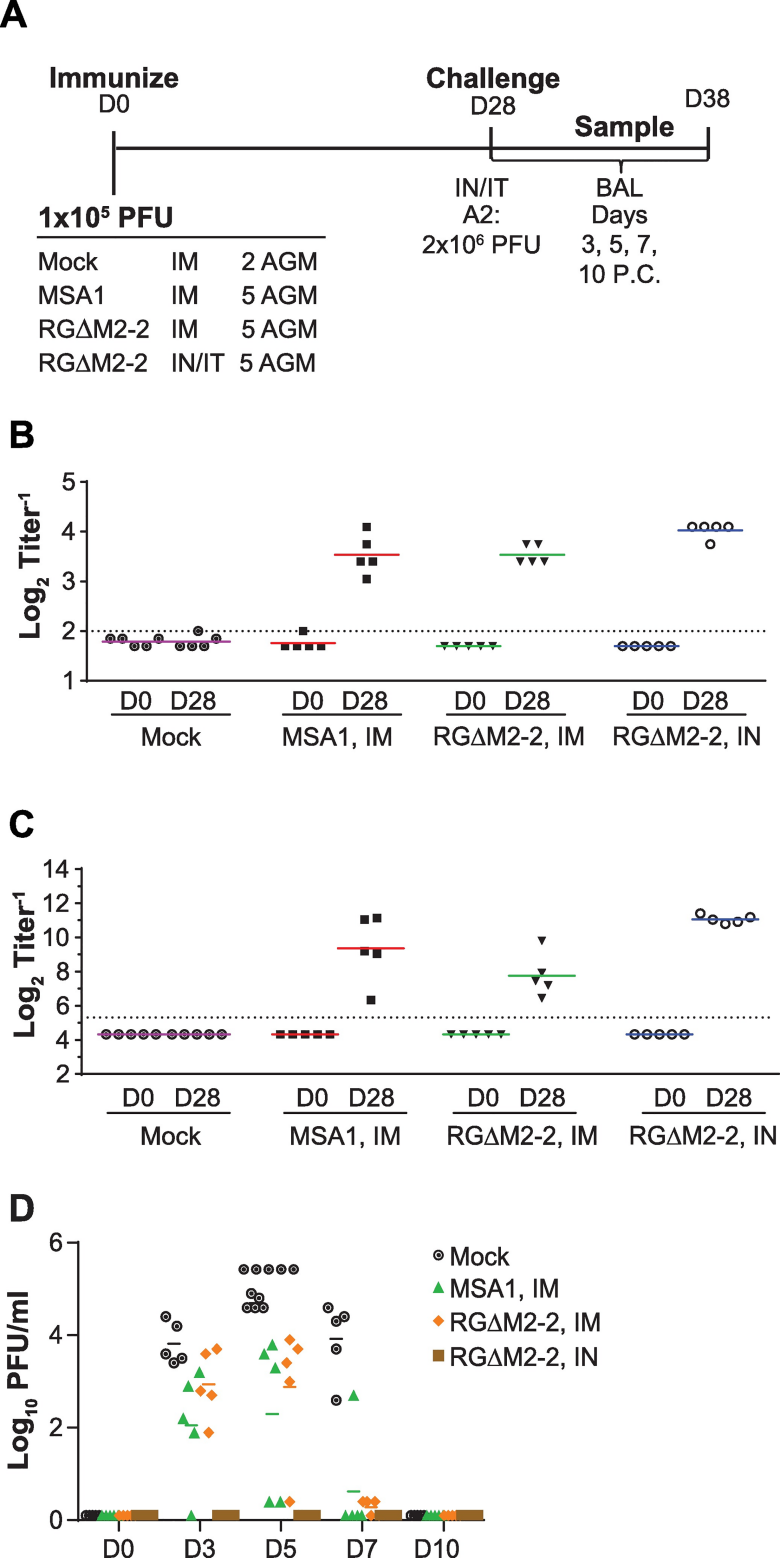
IN vs IM immunization in NHP. (A) AGMs were infected as indicated. (B) Serum F-binding antibodies were determined by ELISA. (C) Complement-dependent serum neutralizing titers were measure by PRNT60. (D) Protective efficacy determined as virus titers from BAL samples after challenge.

Though the RGΔM2-2 candidate was not attenuated in AGMs, we wanted to better understand its immunogenicity and protective efficacy in this model system for different immunization routes. As outlined in Fig 5A, groups of 5 AGMs were either intranasally/intratracheally (IN/IT) or intramuscularly immunized with 10^5.5^ PFU of RSV MSA1 or RGΔM2-2. Twenty-eight days following immunization, all animals were challenged IN/IT with 2x10^6^ PFU RSV A2. BAL samples were taken and titrated on days 3, 5, 7 and 10 post challenge. Serum samples from all animals were taken on D0 before immunization and D28 prior to challenge.

All animals in the MSA1 and RGΔM2-2 groups showed an increase in serum IgG F ELISA titers 28 days after immunization (Fig 5B). These values were slightly higher for the RGΔM2-2 IN/IT immunized group versus the IM immunized groups. Similarly, serum neutralization titers were higher for the RGΔM2-2 IN/IT immunized group over either IM immunized group, MSA1 or RGΔM2-2 (Fig 5C). When BAL samples taken after challenge were analyzed by titration, all IM immunization groups showed intermediate levels of virus relative to mock immunized samples (Fig 5D). However, the RGΔM2-2 IN/IT immunized animals showed complete protection with no detectable virus in the lower respiratory tract. Overall, immunization via the IM route with either MSA1 RSV or RGΔM2-2 afforded a serum antibody response that was similar, though slightly lower, to those induced via IN/IT immunization yet insufficient to confer complete protection as was seen in IN/IT immunized animals. Though protection was incomplete in IM immunized animals, overall levels of challenge virus were lower and clearance achieved more quickly than mock immunized animals.

**Fig 6:**
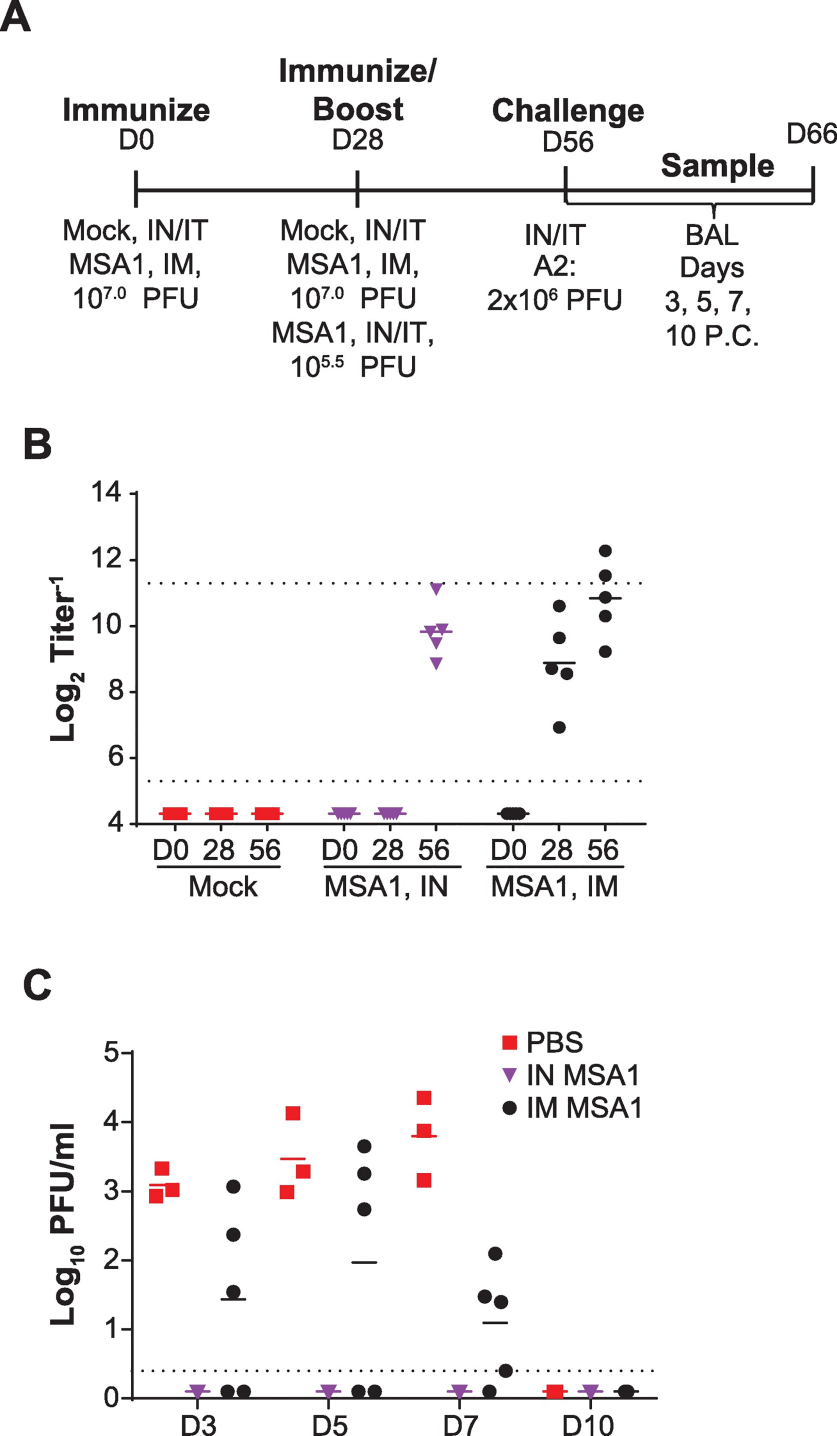
IM boost immunization in NHP. (A) AGMs were infected as indicated. (B) Complement-dependent serum neutralizing titers, PRNT60. (C) Protective efficacy as determined from BAL samples after challenge.

As immunization via the IN/IT route allows for virus replication while the IM route does not, we sought to understand if a boosted immune response could be obtained with a higher IM dose and/or multiple IM immunizations and therefore confer complete protection. One advantage of RSV IM-route vaccination is the ability to potentially boost immunogenicity via higher vaccine dose without concern of attenuation profile. To further analyze this option, we used wildtype RSV to prime and boost five AGMs twenty-eight days apart with 1x10^7^ PFU RSV MSA1 intramuscularly (Fig 6A). An alternative group of 5 AGM was IN/IT immunized with RSV-MSA1 at a 1x10^5.5^ PFU dose on day twenty-eight. On day 56 of the study, all animals were challenged with 2x10^6^ PFU A2 IN/IT. BAL samples were collected for 10 days post challenge. Serum samples were obtained prior to vaccination and prior to challenge. Animals vaccinated either IM or IN/IT showed high levels of serum neutralizing titers on day 56, just prior to challenge (Fig 6B). In animals vaccinated IN/IT, neutralizing titers were 9.8log_2_ on day 56, 28 days after vaccination. IM immunized animals also had high levels of neutralizing titers on day 56 (10.8log_2_). However, despite having high levels of serum neutralizing antibodies, IM immunized animals were not fully protected from challenge (Fig 6C). Primates vaccinated and boosted with 10^7^ PFU RSV IM had intermediate levels of challenge virus in their lungs (2.0log_10_ PFU/ml, peak D5 PC) as compared to undetectable levels in IN/IT immunized animals. While this level is lower than unvaccinated control animals (3.8log_10_ PFU/ml, peak D7 PC), full protection was not achieved despite robust serum neutralizing antibody being present prior to challenge.

## Discussion

We created a live RSV vaccine candidate, RGΔM2-2, by deleting the M2-2 gene from the MSA1 clinical RSV isolate. When this virus was used to immunize animals IM, a potent serum neutralizing antibody response was generated in both primates and cotton rats. This response was sufficient to provide sterilizing immunity upon RSV challenge in the cotton rat model. However, we did not observe complete protection in the lower respiratory tract of non-human primates despite serum antibody titers that would typically be considered protective. This same RGΔM2-2 virus, when administered IN to non-human primates, was able to induce an equally potent serum neutralizing antibody response and confer full protection in the lower respiratory tract. We surmise this difference in protection in the respiratory tract is due to the induction of local mucosal immunity by IN, but not IM immunization.

Our findings in this report also have important implications for preclinical safety testing of live-attenuated vaccine candidates. We initially incorporated a luciferase reporter in our live-attenuated vaccine candidates, and we were able to show conclusively that IM administration resulted in localized protein expression without spreading systemically. This is an important safety consideration for systemic delivery of a replication competent virus. Though an attenuated RSV vaccine is not critical for the IM vaccination approach, we tested the attenuation profile of the RGΔM2-2 vaccine created in a clinical RSV strain background (MSA1). It has been shown that deletion of the M2-2 gene from the A2 strain resulted in a virus that was highly attenuated in the lower respiratory tract of chimpanzees, African green monkeys and cotton rats [25,27,28,32]. Gene deletion of M2-2 has been shown to cause attenuation in other strain backgrounds and also increase protein expression, a feature which may lead to increased immunogenicity of a vaccine [11,12]. In cotton rats, the RGΔM2-2 vaccine was attenuated by 2.9 log_10_ PFU/ml as compared to the parental strain. However, this virus was not attenuated when tested in African green monkeys, nor was attenuation observed when the virus was tested in an *ex vivo* HAE system. The lack of attenuation of RGΔM2-2 suggests the attenuation phenotype associated with deletion of M2-2 is dependent on sequences in the A2 background that are not found in the MSA1 background.

Additionally, the observed discrepancy in the attenuation phenotype between models may also reflect the inherent reliability of these animal models for addressing this question. It is well known that there are host-specific factors that play a role in virus tropism and differences in the attenuation profile of RSV vaccine candidates when tested in different model systems have been seen previously. For example, an A2 strain vaccine bearing a deletion of the SH gene grew to similar titers as wildtype A2 strain in the lungs of mice but showed a 1.6 log_10_ mean peak titer reduction in chimpanzees [6,33]. While the most extensively studied preclinical models of RSV infection, cotton rats and African green monkey, have been used previously for vaccine candidate evaluation [20,22–24], these models are only semi-permissive for RSV replication [34], making accurate preclinical assessment of attenuation difficult. HAE cells may serve as a convenient and accurate *in vitro* test of attenuation, though further evaluation is needed. Prior work has shown that the HAE system accurately predicted the attenuation phenotype of a select panel of RSV vaccine candidates in children [31]. Taken together, these data suggest HAE cells should play a prominent role in assessing the preclinical attenuation phenotype of RSV vaccine candidates. Further comparison of HAE attenuation profiles with additional vaccine candidates are in progress and will contribute to the development of this model for preclinical attenuation evaluation.

It appears induction of mucosal responses is an important consideration for RSV vaccine development. Our results show that though IM immunization yielded potent serum neutralizing responses, it still did not confer full lower respiratory tract protection to challenge in the AGM model. Indeed, it has been reported mucosal IgG/IgA levels correlate better with protection against disease than serum neutralizing titers [35,36]. This is also supported by the finding Synagis, the prophylactic antibody given to high risk infants, can reduce RSV hospitalizations but does not prevent infection [37].

In conclusion, the benefit of improved safety that is obtained through IM delivery of a live-attenuated RSV vaccine is negated by reduced potency, and IN delivery remains the more attractive vaccination strategy. We are currently evaluating novel intranasal live-attenuated vaccine candidates in an ongoing partnership with the National Institutes of Health (NIH). The candidates currently being evaluated reflect refinements in genome sequence composition in an effort to achieve the correct balance of attenuation and immunogenicity. Clinical evaluation of these promising vaccine candidates in naïve infants is underway.
